# The Bioreduction of Selenite under Anaerobic and Alkaline Conditions Analogous to Those Expected for a Deep Geological Repository System

**DOI:** 10.3390/molecules24213868

**Published:** 2019-10-27

**Authors:** Miguel Angel Ruiz-Fresneda, Jaime Gomez-Bolivar, Josemaria Delgado-Martin, Maria del Mar Abad-Ortega, Isabel Guerra-Tschuschke, Mohamed Larbi Merroun

**Affiliations:** 1 Department of Microbiology, University of Granada, 18071 Granada, Spain; jagobo@correo.ugr.es (J.G.-B.); josemariadm93@gmail.com (J.D.-M.); merroun@ugr.es (M.L.M.); 2 Centro de Instrumentación Científica (CIC), University of Granada, 18071 Granada, Spain; mmabad@ugr.es (M.d.M.A.-O.); iguerra@ugr.es (I.G.-T.)

**Keywords:** *Stenotrophomonas bentonitica*, selenite, reduction, alkaline, anaerobic, disposal

## Abstract

The environmental conditions for the planned geological disposal of radioactive waste —including hyper-alkaline pH, radiation or anoxia—are expected to be extremely harsh for microbial activity. However, it is thought that microbial communities will develop in these repositories, and this would have implications for geodisposal integrity and the control of radionuclide migration through the surrounding environment. Nuclear waste contains radioactive isotopes of selenium (Se) such as ^79^Se, which has been identified as one of the main radionuclides in a geodisposal system. Here, we use the bacterial species *Stenotrophomonas bentonitica*, isolated from bentonites serving as an artificial barrier reference material in repositories, to study the reduction of selenite (Se^IV^) under simulated geodisposal conditions. This bacterium is able to reduce toxic Se^IV^ anaerobically from a neutral to alkaline initial pH (up to pH 10), thereby producing elemental selenium (Se^0^) nanospheres and nanowires. A transformation process from amorphous Se (a-Se) nanospheres to trigonal Se (t-Se) nanowires, through the formation of monoclinic Se (m-Se) aggregates as an intermediate step, is proposed. The lesser solubility of Se^0^ and t-Se makes *S. bentonitica* a potential candidate to positively influence the security of a geodisposal system, most probably with lower efficiency rates than those obtained aerobically.

## 1. Introduction

Today, there is a growing concern about the extensive use of nuclear technology due to an increasing radioactive waste inventory. The total amount of nuclear waste—extremely hazardous to the environment and living organisms—may increase in the near future with the potential development of the next generation of nuclear reactors. For this reason, the deep geological repository (DGR) of intermediate and high-level waste (ILW and HLW) has been adopted by many countries for safe storage and isolation [1]. Radioactive residues will accordingly be deposited in steel, iron, or concrete containers and then backfilled with bitumen, a cement matrix, or bentonite clays at a depth of 500–1000 m, in different host rocks depending on the country. Clay formations are to play a crucial role in many DGR designs as host rock or engineered-barriers in France, Belgium, and Switzerland [2]. Specifically, bentonite clays will be employed for the mechanical, hydraulic, and thermal protection of the containers owing to their physical and geochemical properties [3]. It is believed that aerobic conditions will prevail in a DGR system after closure, since oxygen is sure to enter during construction and operational periods. In the post-closure period, anaerobic conditions are likely to be established, with a wide range of electron acceptors, organic matter, and other compounds available. In addition, hyperalkaline (~pH 12) conditions will dominate due to the extensive use of cementitious materials [4].

The environment within a DGR system is estimated to be extremely harsh for living organisms. The presence of radionuclides, high-radiation levels, and limited space for microbial colonization, in addition to the above mentioned anaerobic and hyperalkaline conditions, would restrict microbial viability and activity. However, the capacity of many microorganisms to colonize similarly extreme environments, e.g., hyper-alkaline spring waters or hydrothermal vents, is well known [5,6]. Indeed, it is assumed that different geochemical processes, such as the degradation of cellulose from packaging materials, disposable clothing, and surface wipes present in ILW, can lead to the generation of nutrients and growth substrates that might support the growth of microorganisms. The restoration of water after a thousand years could result in the release of diverse chemical compounds from bitumen and concrete materials used in some DGR systems, including organic matter, electron donors and acceptors (NO_3_^−^), gases (H_2_), etc. The utilization of electron donors and acceptors by anaerobic microorganisms could lead to enzymatic reduction and, therefore, the immobilization of radionuclides like U^VI^, Tc^VII^, Np^V^, or Se^IV^ within the geodisposal system [7,8]. It has been reported that selenium (Se) is present in the tetravalent oxidation state (+IV) in high-level radioactive waste [9]. Water-soluble and toxic Se compounds commonly exist in the oxidation states +IV and +VI as selenite (SeO_3_^2−^) and selenate (SeO_4_^2−^), respectively, while insoluble and harmless Se compounds occurs in oxidation state 0 as elemental Se [10]. The microbial reduction of Se^IV^ has been reported under both aerobic and anaerobic conditions in a wide range of microorganisms. For example, some bacterial strains, such as *Stenotrophomonas maltophilia* SeITE02, *Comamonas testosteroni* S44, and *Bacillus cereus*, can aerobically reduce Se^IV^ to Se^0^ [11,12,13]. Se^IV^ can also be biotransformed anaerobically to Se^0^ by certain bacteria, such as *Azoarcus* sp. CIB, *S. maltophilia*, and *Shewanella oneidensis* MR-1 [14,15,16]. Thus, it is important to study the impact of microbial processes in the transport and mobility of Se through the geodisposal system under conditions relevant to a DGR (aerobic, anaerobic, alkaline conditions, etc.).

The aim of the present work is to investigate the reduction of Se^IV^ by the bentonite-isolated bacterium *S. bentonitica* under anaerobic and alkaline conditions, comparable to those likely to occur in future nuclear waste repositories. In a previous study, we described this interaction under aerobic conditions considering its relevance to nuclear repositories [17]. Specifically, we demonstrated the aerobic bioreduction of Se^IV^ to different amorphous and crystalline Se^0^ nanostructures. Amorphous Se (a-Se) nanostructures are apparently transformed to Se crystals through the role of organic matter. The previous results suggested that *S. bentonitica* would decrease the solubility, and hence the mobility, of Se in the environment surrounding DGRs. Only few papers, however, describe the impact of microbial processes on radionuclide mobility under alkaline conditions analogous to the DGR system [18,19].

To the best of our knowledge, ours is the first study describing the bioreduction of Se^IV^ to Se^0^ anaerobically under different initial pH conditions (from pH 7 to 10) by means of a bacterial strain isolated from Spanish bentonites (Almería, Spain) and selected for DGRs because of their advantageous properties [20]. Flow cytometry studies clearly show that anoxia and Se^IV^ stress negatively affect bacterial viability and activity; although minor viability and activity levels were detected, no cell proliferation was found under the prevailing conditions. In-depth analysis by electron microscopy showed the production of individual and aggregated Se nanospheres and nanowires as reduction products after the Se-bacteria interaction under anaerobic and alkaline conditions (initial pH 10). The selected-area electron diffraction (SAED) pattern of individual Se nanospheres indicated their amorphous nature. However, Raman spectroscopy equipped to a variable pressure field emission scanning electron microscopy (VP-FESEM) indicated the crystalline structure of the Se aggregates (monoclinic Se) and nanowires (trigonal Se), suggesting a transformation process from amorphous to crystalline Se. Not only the oxidation state but also the shape and the structure of the Se reduction products can influence their solubility and mobility. The present study further demonstrates the influence that *S. bentonitica* could have on future DGR systems by reducing the toxicity and mobility of Se^IV^ under an anoxic and high-pH environment, analogous to those that would develop in the repositories of radioactive waste.

## 2. Results and Discussion

### 2.1. Se^IV^ Reduction under Anaerobic and Neutral pH Conditions

#### 2.1.1. Se^IV^ Reduction and Growth Profile

The growth and Se^IV^ reduction of *S. bentonitica* under anoxic conditions was evaluated in the presence of a wide range of added electron donors (sodium acetate, citrate, pyruvate, etc.) and acceptors (sodium nitrate, iron(III) hydroxide, ferric citrate, etc.) at neutral pH conditions in an R2A* medium (a modified composition). The literature data show a great number of compounds that can be used by many microorganisms as electron donors or acceptors in reducing Se^VI^ and Se^IV^. A high reduction rate of Se^IV^ was achieved by *Veillonella atypica* when hydrogen (H_2_) was used as the electron donor under anaerobic conditions, but no reduction was seen when the cells were supplemented with acetate or formate as an electron source [21]. Meanwhile, Kessi and Hanselmann [22] hypothesized that reduced glutathione (GSH) functions as the main electron donor reacting with Se^IV^ in *Rhodospirillum rubrum* and *Escherichia coli*. The possible role of GSH and other reactive thiol compounds in Se^IV^ reduction was likewise suggested within the genus *Stenotrophomonas* by *S. maltophilia* SeITE02 [10]. Other electron sources, such as acetate, lactate, formate, and pyruvate, have been put forth as electron donors in the Se^IV^ reductions in *Geobacter sulfurreducens*, *Shewanella* sp. HN-41, and *S. oneidensis* MR-1 [16,23,24,25]. Se^IV^ acts as the terminal electron acceptor for many microorganisms [26]. However, the use of iron (Fe^III^), nitrate (NO_3_^−^), nitrite (NO_2_^−^), or sulphite (SO_3_^2−^) as electron acceptors by respiratory reductases might support the reduction of Se^IV^ in some microorganisms [27,28]. In the present study, the highest Se^IV^ reduction efficiency—quantified as red precipitate production—by the cells of *S. bentonitica* was observed when sodium acetate and nitrate were added.

Both acetates and nitrates are compounds that will be present in the geodisposal system of radioactive waste. Among other sources, acetate can derive from the oxidation of glucose and degradation of the phthalic acid esters (PVC plasticisers) characteristic of ILW, while nitrates are mainly contained within bitumen waste or as a product of the nitrogen biogeochemical cycle [23,24,25,26,27,28,29,30,31]. The presence of acetate and nitrates within the repositories could thus support the reduction of Se^IV^ by the cells of *S. bentonitica* when anaerobic conditions prevail. For this reason, they were selected for the rest of the experiments as electron donor and acceptor sources, respectively. In addition, Se^IV^ reduction was observed when anaerobically incubating the *S. bentonitica* in the PIPES buffer without the addition of an electron donor or acceptor, suggesting that Se^IV^ could act as the terminal electron acceptor.

**Figure 1:**
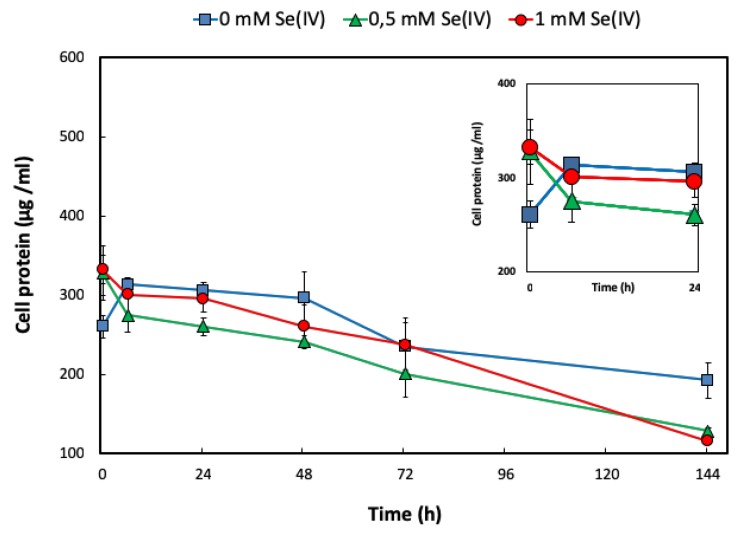
Anaerobic growth curves of *S. bentonitica* in an R2A* medium with sodium acetate and sodium nitrate in the presence of different Se^IV^ concentrations (0 to 1 mM). The inset graph shows a magnification of the growth curve in the first 24 h. Data are presented as the averages ± standard errors.

In the absence of Se^IV^, only when the cultures were amended with sodium acetate and sodium nitrate was a limited increase in the total cell protein observed during the first 12 h of incubation (Figure 1). Notwithstanding, no growth was detected in the presence of Se^IV^ at any concentration tested (0.5 and 1 mM) (Figure 1), despite the fact that previous studies revealed the capacity of this bacterium to grow aerobically under Se^IV^ stress [17]. Such results point to a toxic effect of Se^IV^ on *S. bentonitica* cells under anaerobic conditions. Nevertheless, the red precipitates produced in all the cultures amended with Se^IV^ clearly indicate the ability of this bacterium to anaerobically reduce Se^IV^ to Se^0^. The non-production of Se red precipitates in Se^IV^-untreated cultures and Se^IV^-treated media (abiotic controls) confirmed Se^IV^ reduction as a biological process.

#### 2.1.2. Cell Viability and Metabolic Activity

The effect of Se^IV^ on the cell viability of *S. bentonitica* was studied by means of the live–dead staining approach, conducted with propidium iodide (PI) and fluorescein diacetate (FDA). PI enters into cells with damaged membranes, staining the nucleic acids of dead cells [32], whereas FDA stains viable cells [33]. To test metabolic activity, the fluorescent dye DiOC_6_ was used in order to bind the polarized membranes of the active cells [34].

**Figure 2:**
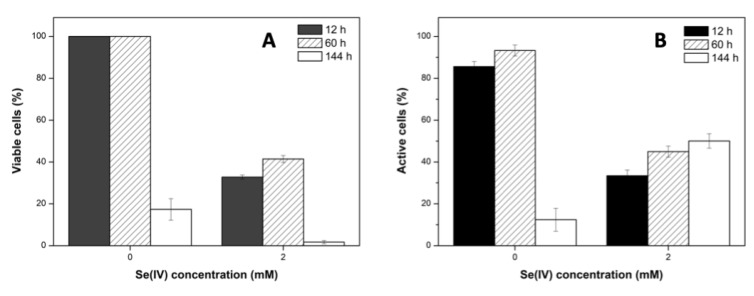
Percentage of viable (**A**) and active cells (**B**) of *S. bentonitica* under different Se^IV^ concentrations (0 and 2 mM) and contact times (12, 60, and 144 h) under anaerobic and neutral pH conditions.

The percentages of viable and active cells of *S. bentonitica*, both untreated and treated with 2 mM Se^IV^ incubated anaerobically at a neutral pH from 12 h to 144 h, are displayed in Figure 2. Cell viability was negatively affected by Se^IV^. Specifically, 32.7%, 41.5%, and 1.7% of cell populations were found to be viable after 12, 60, and 144 h of incubation, respectively, in the presence of 2 mM Se^IV^. In contrast, higher viable cell populations (100%, 100%, and 17.3%) were observed in untreated samples at the same incubation times. Our previous studies under aerobic conditions gave higher viability values for the cells after 144 h of incubation in the presence of 2 mM Se^IV^ (unpublished data). These results underline the influence of anoxia and Se^IV^ on the viability of *S. bentonitica* cells. On the other hand, the metabolic activity test generally showed a lower oxidative response under 2 mM Se^IV^ stress (compared with Se^IV^-untreated cells) at all times assayed, except at 144 h. Specifically, 33.4%, 45%, and 50% were found to be active after 12, 60, and 144 h at 2 mM Se^IV^ stress, while 85.6%, 93.3%, and 12.4% of the cells were active in untreated samples. The increases in cell activity after 144 h in the presence of Se^IV^ compared to the Se^IV^-untreated cells might be explained by the cellular metabolic response to Se^IV^ stress. Our previous studies under aerobic conditions showed similar behavior (unpublished data).

### 2.2. Se^IV^ Reduction under Anaerobic and Alkaline Conditions

**Figure 3:**
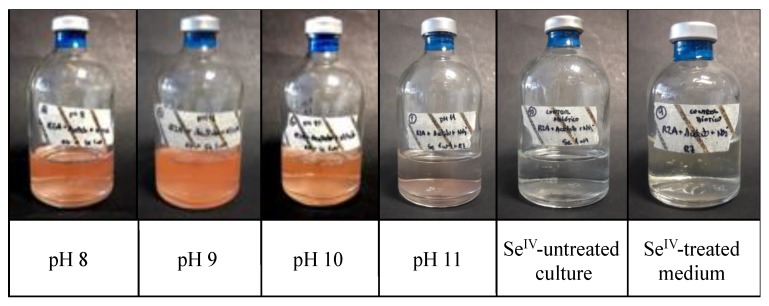
Cultures of *S. bentonitica* adjusted to pH 8, 9, 10, and 11 showing the reduction of 2 mM Se^IV^ from pH 8 to 10, as indicated by their reddish colour. Se^IV^-untreated cultures and the Se^IV^-treated medium were employed as controls.

Se’s reduction ability was also studied under anaerobic and alkaline initial pH conditions analogous to those expected in a DGR environment. The colour of the Se^IV^-treated cultures (2 mM) of *S. bentonitica* turned reddish at initial pH 8, 9, and 10 after 24/48 h of incubation (Figure 3). However, no red precipitates were observed at pH 11, probably due to the non-proliferation of the cells under these conditions. The latter hypothesis is supported by previous investigations indicating that *S. bentonitica* grows at pH 5–10 under aerobic conditions [35]. As described for the anaerobic experiments in Section 2.1., no colour change in the Se^IV^-untreated cultures and Se^IV^-treated media confirmed the bioreduction of Se^IV^. Many bacterial strains are known to reduce Se^IV^ anaerobically [14,16]. However, to the best of our knowledge, this is the first study describing the microbial reduction of Se^IV^ at an alkaline pH (up to pH 10) under anaerobic conditions. These results reveal the ability of the bentonite isolate *S. bentonitica* to reduce Se toxic forms to non-toxic Se under the analogous conditions expected in a DGR system. Therefore, we propose that this species of the genus *Stenotrophomonas* could contribute to the immobilization of Se within the DGR concept.

**Figure 4:**
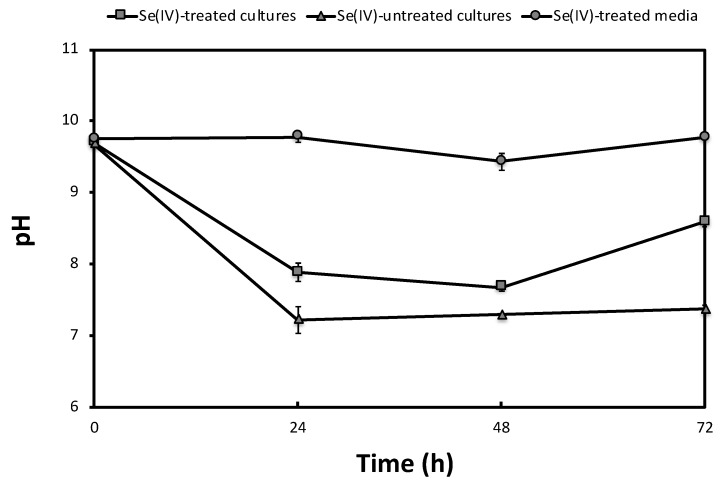
Time course of pH variation in Se^IV^-treated (2 mM) and untreated cultures of *S. bentonitica* adjusted to pH 10 under anaerobic conditions. Se^IV^-treated media were employed as abiotic controls. Se^IV^ and cells were added at zero time. Each curve shows the means based on the results of triplicates.

To study the effect of Se and the cells on the pH variation of the samples (adjusted to pH 10), the pH was measured at different times during incubation. Abiotic controls (Se^IV^-treated media) showed no variation of the pH over time, remaining around 10 during the entire assay (Figure 4). In the presence of bacteria, however, both untreated and treated cultures of *S. bentonitica* showed a decrease, respectively, from pH 10 to pH 7 and 8, by increasing incubation time (Figure 4). The cells are responsible for this decrease in pH, perhaps as a consequence of the bacterial metabolic activity responding to these stressful conditions, as suggested by the flow cytometry results.

Rizoulis et al. [36] studied the reduction of different electron acceptors of relevance to ILW in natural alkaline sediments set at pH 10, 11, and 12. Similar to our experiments, they noted a slight drop in pH during incubation, probably due to the formation of CO_2_ from the metabolism of lactate, acetate, and other carbon sources present in the sediments. In our case, *S. bentonitica* was able to oxidize acetate to CO_2_, which in turn led to a decreased in the pH of the medium (to 7–8) where Se^IV^ reduction occurs.

### 2.3. Electron Microscopic Characterization of Se^IV^ Bioreduction Products

**Figure 5:**
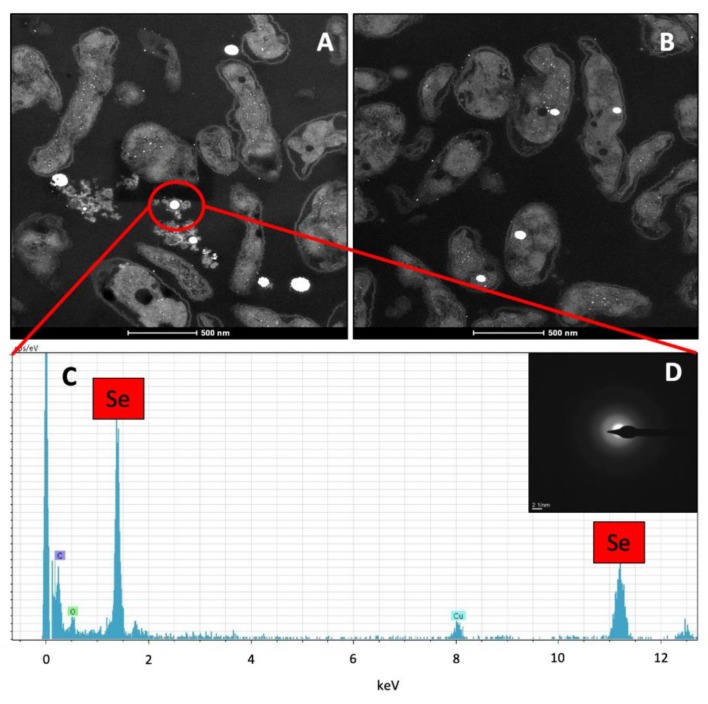
HAADF-STEM analysis of an ultrathin-sectioned *S. bentonitica* sample incubated anaerobically at a neutral pH in the presence of 2 mM Se^IV^ (**A**,**B**). EDX analysis revealed the Se composition of the electron-dense nanospheres (**C**) located both intracellular and extracellularly (**A**,**B**). SAED pattern derived from an individual Se granule (**D**). Scale bars: 500 nm (**A**,**B**).

Ultrathin sections showed electron-dense nanospheres produced by the cells of *S. bentonitica* on the surface and within the intracellular and extracellular space after 144 h incubation on Se^IV^ at a neutral pH (Figure 5A,B). The size of both intracellular and extracellular nanospheres ranged between 100 and 200 nm (Figure 5A,B). EDX microanalysis confirmed their Se composition (Figure 5C). In addition, the SAED pattern derived from a single nanosphere showed an amorphous nature (Figure 5D).

**Figure 6:**
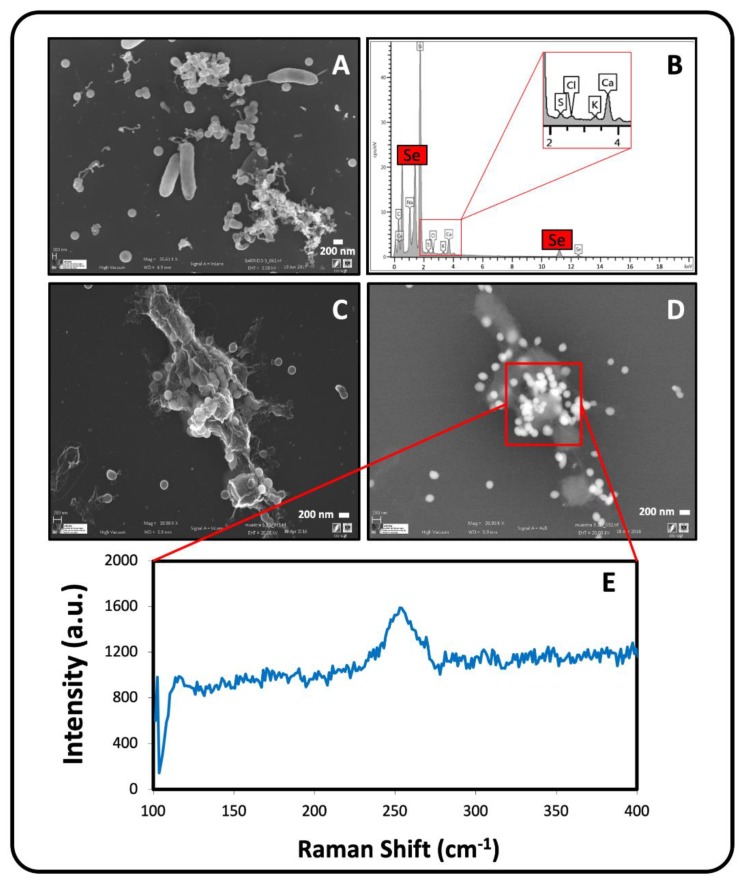
VP-FESEM micrographs of Se nanospheres located extracellularly, intracellularly and attached on lysed cells of *S. bentonitica* (**A**,**C**,**D**) produced anaerobically at an initial pH of 10. EDX analysis showing the Se and S composition of the nanospheres (**B**). Raman analysis derived from Se nanosphere accumulations (**E**). Scale bars: 200 nm (**A**,**C**,**D**).

Likewise, we noted the presence of individual Se nanospheres located extracellularly, intracellularly, and attached on the cell surface after 144 h of anaerobic incubation at an initial pH of 10 by using a VP-FESEM system (Figure 6A–D). In addition to Se, EDX microanalysis revealed the presence of S (Figure 6B), suggesting the relevant contribution of thiol-containing biomolecules, such as GSH in the reduction pathway of Se^IV^. The Se nanospheres formed aggregates (Figure 6C,D), as observed in aerobic cultures of *S. bentonitica* treated with Se^IV^ [17]. The formation of Se aggregates by *S. bentonitica* could lead to the transformation of amorphous Se (a-Se) nanospheres to trigonal Se (t-Se) nanostructures with high stability, as reported by Ruiz-Fresneda et al. [17], when this bacterium was grown aerobically. The transformation from a-Se to t-Se is a time-dependent process. The a-Se nanospheres are initially released to the extracellular matrix from the cytoplasm (24 h) and most probably start to form aggregates on the axis of flagella-like proteins after 48–72 h. The aggregation of Se nanoparticles (SeNPs) using proteins as a template may play a crucial role in the transformation and crystallization mechanism [17,37]. Finally, after 144 h, the aggregates crystallize, producing t-Se nanostructures of different shapes (nanowires, hexagons, and polygons) and sizes. However, further investigations are needed to confirm that a transformation process occurs under conditions such as those employed in the present study.

**Figure 7:**
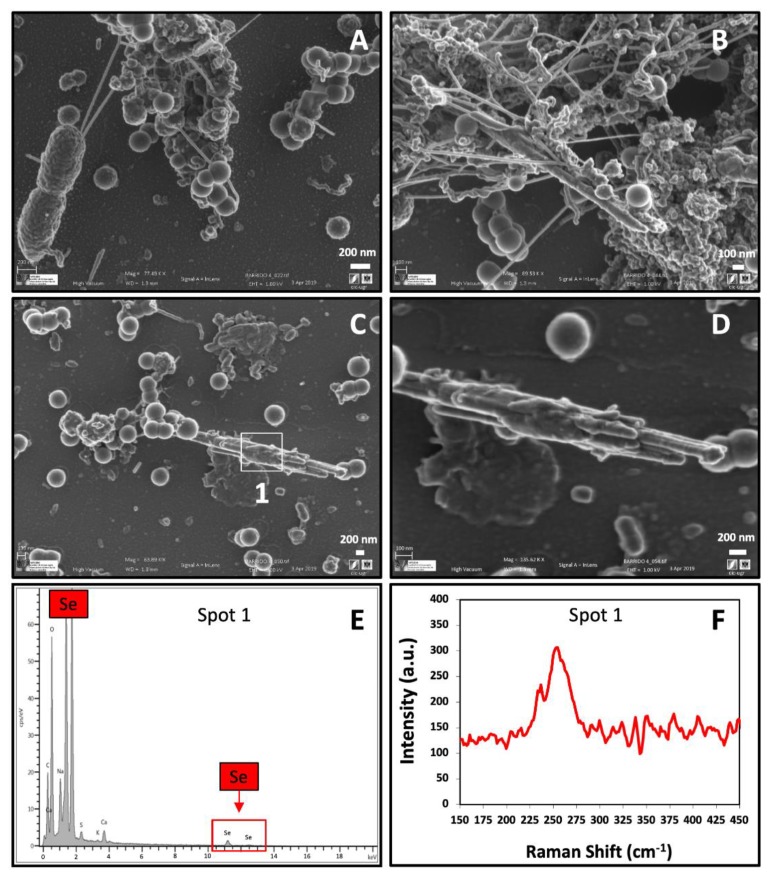
VP-FESEM micrographs showing SeNPs associated with organic materials and filaments (**A**,**B**) and Se nanowires within the extracellular space (**C**,**D**) under anaerobic conditions at an initial pH of 10. EDX analysis of an individual nanowire indicates its Se composition (**E**). Raman scattering spectrum derived from the same nanowire (**F**). Scale bars: 200 nm (**A**,**C**,**D**) and 100 nm (**B**).

The Raman scattering spectrum derived from the observed Se nanosphere accumulations exhibited an intense peak at 254 cm^−1^ (Figure 6E). Kora and Rastogi [38] suggested that a resonance peak at 254 cm^−1^ could be attributed to a-Se as a result of Se atoms being irregularly arrayed in disordered chains. These findings contrast with those previously obtained for the SeNPs synthesized by *S. bentonitica* under aerobic conditions, where a peak at 235 cm^−1^ corresponding to t-Se was observed. Other authors indicated that a peak centered at 254 cm^−1^ is characteristic of crystalline monoclinic Se (m-Se) [39,40]. Over the last century, many contradictory studies of the a-Se structure have come to light [41]. The three allotropes of m-Se are a deep red colour and have Se_8_ rings as structural units within their unit cell [42]. Amorphous Se (red or black) is thought to contain 2 polymeric chains (Se_n_) and Se_8_ monomeric rings [43,44]. It is, therefore, very complicated to distinguish between a-Se and m-Se structures. The Se aggregates produced by *S. bentonitica* cells could be amorphous or monoclinic, in contrast to the individually distributed Se nanospheres described above as amorphous by means of SAED. However, it is well known that a-Se could be transformed to m-Se nanostructures, which in turn could be transformed to t-Se as one of the most thermodynamically stable phases [37,45]. Accordingly, the formation of m-Se aggregates by *S. bentonitica* may be an intermediate thermodynamic step during the possible transformation to t-Se. A great abundance of organic matter and organic filaments probably corresponding to flagella-like proteins were detected surrounding SeNPs and bacterial cells (Figure 7A–B). The VP-FESEM images clearly show that the SeNPs embedded in this organic matrix are mainly composed by proteins, suggesting their role in aggregation and transformation.

**Figure 8:**
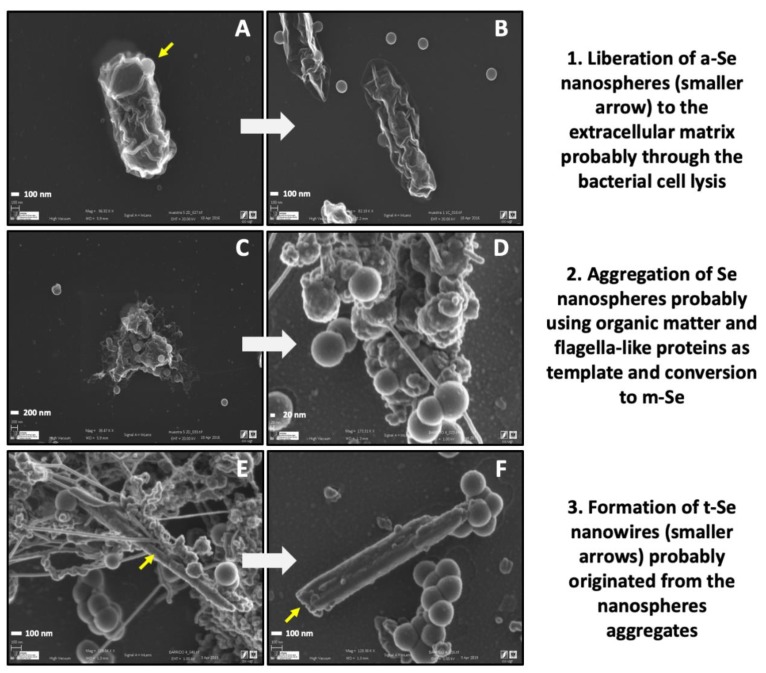
VP-FESEM images representing the proposed transformation from a-Se nanospheres to t-Se nanowires through the formation of m-Se aggregates as an intermediate step by using proteins as a template. The images correspond to samples prepared by growing *S. bentonitica* anaerobically at an initial pH of 10. Scale bars: 100 nm (**A**,**B**,**E**,**F**), 200 nm (**C**), and 20 nm (**D**).

Finally, few extracellular Se nanowires could be detected in the proximity of Se aggregates (Figure 7B–D). The Raman scattering spectrum derived from the nanowires displayed two main peaks, one at 236 cm^−1^ and 254 cm^−1^ (Figure 7F). The resonance peak at 236 cm^−1^ could be linked to t-Se, while 254 cm^−1^ could be attributed, as mentioned before, to the intermediate thermodynamic phase, a-Se or m-Se. This suggests that a transformation process could take place, likely along the lines reported by Ruiz-Fresneda et al. [17], including several steps. The intracellular Se nanospheres would be released through the bacterial cell lysis, as indicated by the seriously damaged cells observed (Figure 8). Afterwards, they formed aggregates within the extracellular matrix, probably using cellular organic matter and flagella-like proteins as a template (Figure 8). Finally, the Se nanowires appear to originate from the accumulation and aggregation of the nanospheres attached to these organic matter and protein filaments (Figure 8). In the proposed mechanism, the individual a-Se nanospheres would crystallize to m-Se aggregates and t-Se nanowires.

**Figure 9:**
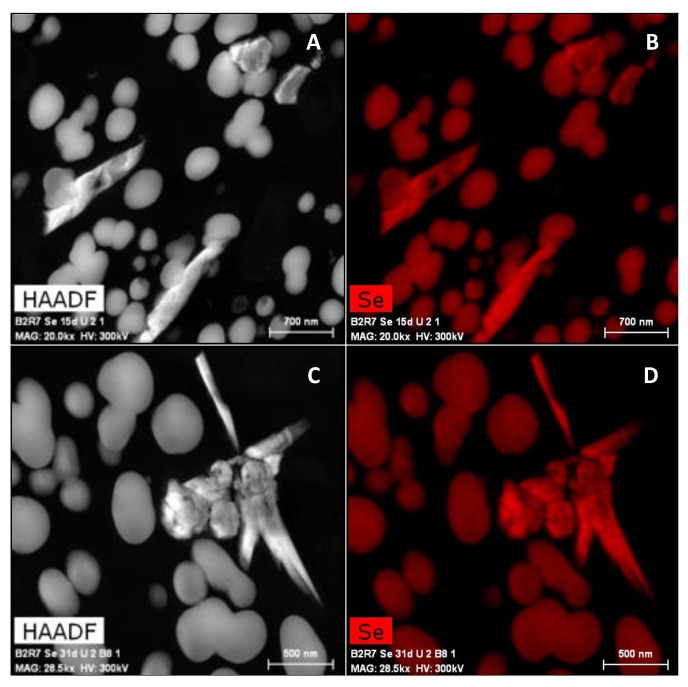
HAADF-STEM micrographs of Se nanostructures aerobically produced by *S. bentonitica* in the presence of 2 mM Se^IV^ for 15 days (**A**–**D**). Corresponding elemental maps showing the Se composition of the nanostructures. Scale bars: 700 nm (**A**,**B**) and 500 nm (**C**,**D**).

The small number of nanowires observed in comparison with our previous studies conducted under oxic conditions [17] points to the likelihood that under anaerobic and alkaline conditions, the cells need longer incubation times to produce Se crystals. A microscopic analysis of samples aerobically incubated supports this hypothesis, with a greater presence of Se crystals over time, from 15 to 30 days (Figure 9). This transformation is likely to occur more slowly because of the harsh conditions to which the cells were exposed. These conditions influenced the cell viability and proliferation, as revealed by flow cytometry, and would have also affected the processes of their reduction and transformation to Se crystals.

The reduction from Se^IV^ to Se^0^ under conditions simulating DGR (anaerobic and alkaline) would have a clearly positive impact onto the safety of these systems, due to the lower toxicity and mobility of Se at a zero valent oxidation state. However, the different Se structures and shapes produced must also be taken into account when predicting the specific influence of *S. bentonitica* cells. It is generally assumed that SeNPs are insoluble and non-toxic [40]. In fact, microbially produced Se nanospheres have been shown to be less toxic than oxidized Se species (Se^VI^ and Se^IV^) [46]. Thus, the production of Se nanospheres by *S. bentonitica* would be beneficial within a geodisposal system. Notwithstanding, there are considerable uncertainties about the toxicity of SeNPs compared to their soluble oxidized forms, according to some studies [47,48]. Li et al. [48] described a greater toxicity of SeNPs in comparison with Se^IV^ in the fish *Oryzias latipes*. Kumar et al. [47] reported that the toxicity of SeNPs depends on their concentration, in this case documenting toxic effects on the fish species *Pangasius hypophthalmus*. Further research is needed to arrive at conclusive evidence for the toxicity level of SeNPs produced by *S. bentonitica*. The formation of crystalline Se in the form of nanowires could have a positive impact on the repositories, as suggested by their lower mobility and higher settleability when compared to Se nanospheres and oxyanions [49,50]. The lesser volume of Se crystalline nanowires observed indicates a lower short-term efficiency of *S. bentonitica* in the immobilization of the Se^IV^ present in the repositories when anaerobic and alkaline conditions dominate, as opposed to aerobic studies [17].

## 3. Materials and Methods

### 3.1. Bacterial Strain and Growth Conditions

The bacterial strain *S. bentonitica* employed in the present study was isolated from Spanish bentonites collected in Cabo Gata Nature Park (Almeria, Spain) [51]. The bacterial cells were grown aerobically in a solid and liquid Luria-Bertani (LB) broth medium (tryptone 10 g/L, yeast extract 5 g/L and NaCl 10 g/L, pH 7.0 ± 0.2) at 28 °C. The *S. bentonitica* cells were inoculated to an initial optical density (O.D.) of 0.2 (at 600 nm) for all the experiments.

### 3.2. Anaerobic Growth under Selenite Stress

*S. bentonitica* were grown in a degassed R2A* (modified composition: peptone 0.5 g/L, glucose 0.5 g/L, K_2_HPO_4_ 0.3 g/L, and MgSO_4_ 0.05 g/L) medium, to which we added with different electron donors (sodium acetate, citrate, pyruvate, etc.) and acceptors (sodium nitrate, iron (III) hydroxide, ferric citrate, etc.) in order to determine their ability to grow anaerobically. Cells were harvested at the mid-exponential phase by centrifugation (10000× g; 10 min) from LB cultures and washed with 30 mM PIPES buffer to remove the medium’s ingredients. Afterwards, the cells were re-suspended in R2A* supplemented with the corresponding electron donor and acceptor. An R2A* medium is commonly used to isolate bacteria from oligotrophic environments, including those from radioactive waste repositories [52,53]. This culture medium is, therefore, appropriate to study the influence of microbial processes on the mobility of radionuclides in the context of a DGR system. The component peptone was employed as a nitrogen and undefined electron donor source needed for the growth and the Se reduction processes. Acetate was added in addition to peptone since *S. bentonitica* uses just a few carbon sources and electron donors (acetate, gluconate) [35]. Se^IV^ at different concentrations (0.1 to 1 mM) was added to the mixture from a 1M sodium selenite (Na_2_SeO_3_) (Sigma-Aldrich, St. Louis, MO, USA) stock solution. The solubility of Se^IV^ in this medium (R2A*) is not affected by its components, as demonstrated in our previous work with a more complex medium (LB-Luria Bertani) [17].

Finally, the suspensions were degassed using N_2_ and incubated at 28 °C. Se^IV^-untreated cells and an R2A* medium containing Se^IV^ were used as controls. All the measurements were performed in triplicate. Growth was determined by quantifying the total protein content in the bacterial cell extracts using a modification of the method described by Dhanjal and Cameotra [54]. A 1 mL aliquot of bacterial culture was taken at different time intervals to measure growth, based on the protein content, using the Bradford reagent (Bio-Rad^TM^) [55]. Bovine serum albumin (BSA) was used as a standard.

### 3.3. Reduction of Se^IV^ under Anaerobic and Alkaline Conditions

The Se^IV^ reduction ability of *S. bentonitica* was also assayed anaerobically under alkaline conditions (from pH 8 to 11) relevant for the DGR system. The samples were prepared as described in the section above (Section 3.2.), using sodium acetate and sodium nitrate as the electron donor and acceptor, respectively. The pH of the solutions was adjusted by the addition of acid (HClO_4_) or base (NaOH) using a pH-meter (CRISON^©^ micro pH 2002, Barcelona, Spain), and the samples were sterilized by filtration through 0.22 µm nitrocellulose filters prior to degassing.

### 3.4. Flow Cytometry

The cell viability and the metabolic activity of *S. bentonitica* in the presence of Se^IV^ under anoxic conditions were determined by means of the flow cytometry technique. For this purpose, the cultures were prepared as described in Section 3.2 in the presence of acetate and nitrate, with an initial concentration of Se^IV^ 2 mM. All experiments were conducted in triplicate. After 12, 60, and 144 h of incubation, the cells were collected by centrifugation at 11000× *g* and 4 °C for 10 min. The resultant pellet was washed four times in phosphate buffered saline (PBS) with pH 7. The cells were then dissolved in PBS, adjusting their cellular density to approximately 10^6^ cells/mL. For the cell viability test, fluorescein diacetate (FDA) and propidium iodide (PI) were added to each sample to final concentrations of 20 μL/mL and 2 μL/mL, respectively. To perform the metabolic activity test, 3,30-dihexyloxacarbocyanine iodide (DiOC_6_) was used at a final concentration of 20 μL/mL. Finally, the samples were analysed by Forward Scatter using a FACSCanto II^TM^ cytometer (Becton Dickinson, San Jose, CA, USA). Se^IV^-free cultures and dead cells were obtained by heating the biomass at 90 °C served as controls.

### 3.5. Electron Microscopy

Variable pressure field emission scanning electron microscopes (VP-FESEMs) equipped with an X-ray detector Raman spectroscopy system (SCA-Structural and Chemical Analyser, Gloucestershire, UK) made possible an in situ 3-D structural and elemental characterization of the reduced Se produced by the cells. Se^IV^-amended cultures (2 mM) with the addition of sodium acetate and sodium nitrate, respectively as electron donor and acceptor, were prepared for VP-FESEM after 144 h of anaerobic growth at different pHs (7–10) as described by Ruiz-Fresneda et al. [17]. The samples were analysed under a VP-FESEM Zeiss SUPRA 40VP (Carl Zeiss, Oberkochen, Germany).

The Se reduction products were also analysed by means of a scanning transmission electron microscope (STEM) equipped with energy dispersive X-ray (EDX). EDX analysis was performed at 300 kV using a spot size of 4 Å and a live counting time of 50 s. The structural characterization of Se nanostructures was based on SAED. The samples were prepared as described in Merroun et al. [56] and examined under a high-angle annular dark field scanning transmission electron microscope (HAADF-STEM) FEI TITAN G2 80–300 (FEI Europe, Eindhoven, Netherlands). The STEM specimen holders were cleaned by plasma prior to STEM analysis to minimize contamination.

## 4. Conclusions

The present study describes the ability of bentonite-isolated *S. bentonitica* to reduce Se^IV^ to Se^0^ under anaerobic and alkaline conditions, simulating those expected in a DGR. Despite the low cell viability, low activity, and absence of growth of *S. bentonitica* in the presence of Se^IV^, individual and aggregated Se nanospheres and lower amounts of Se nanowires were produced as a consequence of the bioreduction process. A combination of microscopic and spectroscopic techniques suggested the transformation from a-Se nanospheres to crystalline Se nanowires (t-Se) in a way similar to that reported aerobically by Ruiz-Fresneda et al. [17]. A slower transformation process appears to occur due to the non-proliferation and low viability and activity rates of *S. bentonitica* cells, most likely caused by harsh environmental conditions. However, further studies are needed to confirm the proposed transformation process.

The redox transformation from toxic Se^IV^ to non-toxic Se^0^ and the formation of crystalline Se nanowires suggests *S. bentonitica* to be a potential bacterial species with a positive effect on the safety of the DGR system in the event of a radionuclide escape. However, the slower transformation process suggests the limited short-term efficiency of *S. bentonitica* in the formation of crystalline Se and, hence, in the immobilization of Se when anaerobic and alkaline conditions predominate. More specific studies are needed to determine the mobility of the Se nanospheres produced by this bacterium within a DGR system. Still, the present study contributes to our understanding of the impact of microbial processes on the toxicity of Se in the future disposal of radioactive waste.
